# TCA cycle metabolites associated with adverse outcomes after acute coronary syndrome: mediating effect of renal function

**DOI:** 10.3389/fcvm.2023.1157325

**Published:** 2023-06-27

**Authors:** Raul Sanchez-Gimenez, Óscar M. Peiró, Gil Bonet, Anna Carrasquer, George A. Fragkiadakis, Mònica Bulló, Christopher Papandreou, Alfredo Bardaji

**Affiliations:** ^1^ Department of Cardiology, Joan XXIII University Hospital, Tarragona, Spain; ^2^ Institute of Health Pere Virgili (IISPV), Tarragona-Reus, Spain; ^3^ Department of Medicine and Surgery, Rovira I Virgili University, Tarragona, Spain; ^4^ Department of Nutrition and Dietetics Sciences, School of Health Sciences, Hellenic Mediterranean University, Siteia, Greece; ^5^ Nutrition and Metabolic Health Research Group, Department of Biochemistry and Biotechnology, Rovira I Virgili University, Reus, Spain; ^6^ Center of Environmental, Food and Toxicological Technology – TecnATox, Rovira i Virgili University, Reus, Spain; ^7^ CIBER Physiology of Obesity and Nutrition (CIBEROBN), Carlos III Health Institute, Madrid, Spain

**Keywords:** tricarboxylic acid cycle, metabolomics, mass spectrometry, acute coronary syndrome, major adverse cardiovascular events, mortality

## Abstract

**Aims:**

To examine relationships of tricarboxylic acid (TCA) cycle metabolites with risk of cardiovascular events and mortality after acute coronary syndrome (ACS), and evaluate the mediating role of renal function in these associations.

**Methods:**

This is a prospective study performed among 309 ACS patients who were followed for a mean of 6.7 years. During this period 131 patients developed major adverse cardiovascular events (MACE), defined as the composite of myocardial infarction, hospitalization for heart failure, and all-cause mortality, and 90 deaths were recorded. Plasma concentrations of citrate, aconitate, isocitrate, succinate, malate, fumarate, α-ketoglutarate and d/l-2-hydroxyglutarate were quantified using LC-tandem MS. Multivariable Cox regression models were used to estimate hazard ratios, and a counterfactual-based mediation analysis was performed to test the mediating role of estimated glomerular filtration rate (eGFR).

**Results:**

After adjustment for traditional cardiovascular risk factors and medications, positive associations were found between isocitrate and MACE (HR per 1 SD, 1.25; 95% CI: 1.03, 1.50), and between aconitate, isocitrate, d/l-2-hydroxyglutarate and all-cause mortality (HR per 1 SD, 1.41; 95% CI: 1.07, 1.84; 1.58; 95% CI: 1.23, 2.02; 1.38; 95% CI: 1.14, 1.68). However, these associations were no longer significant after additional adjustment for eGFR. Mediation analyses demonstrated that eGFR is a strong mediator of these associations.

**Conclusion:**

These findings underscore the importance of TCA metabolites and renal function as conjunctive targets in the prevention of ACS complications.

## 1. Introduction

Cardiovascular disease (CVD) and acute coronary syndrome (ACS) are the leading causes of morbidity and mortality worldwide causing a high health cost due to their consequences (1). Prognostic classification in patients admitted with ACS is necessary for providing efficient follow-up and guiding treatment. During the last years, many efforts have been made to identify predictive biomarkers for adverse outcomes following ACS (2) and metabolomics seems a promising approach (3).

Since metabolic perturbations are frequent in ACS (4), tricarboxylic acid cycle (TCA) disturbances could be related to mitochondrial dysfunction, causing oxidative stress and introducing a pro-inflammatory state (5). The role of TCA cycle enzymes and metabolites in various human diseases, i.e., neurologic or tumoral, has received increased attention in recent years (6). Although, their role in cardiovascular development is still uncertain, recently plasma TCA cycle-related metabolites were associated with heart failure and atrial fibrillation in an older population at high CVD risk (7). However, their prognostic role in ACS is unclear.

Furthermore, TCA cycle metabolites are involved in several kidney functions (8, 9), whereas renal function is an important regulator of circulating TCA metabolites levels (10, 11), which raises the question to whether these metabolites are directly associated with risk of adverse clinical outcomes after ACS or if renal function plays potentially mediating role in these associations.

The aim of this prospective cohort study was to investigate the relationships of TCA cycle disturbances as monitored by plasma metabolites, with risk of major adverse cardiovascular events (MACE), and all-cause mortality in ACS patients and examine whether and to what extend these associations are mediated by renal function, assessed by estimated glomerular filtration rate (eGFR).

## 2. Materials and methods

### 2.1. Study design and participants

All patients provided written informed consent to participate in the study. The Institutional Ethical Committee approved the study which was conducted in accordance with the ethical principles of the Declaration of Helsinki.

**Figure 1:**
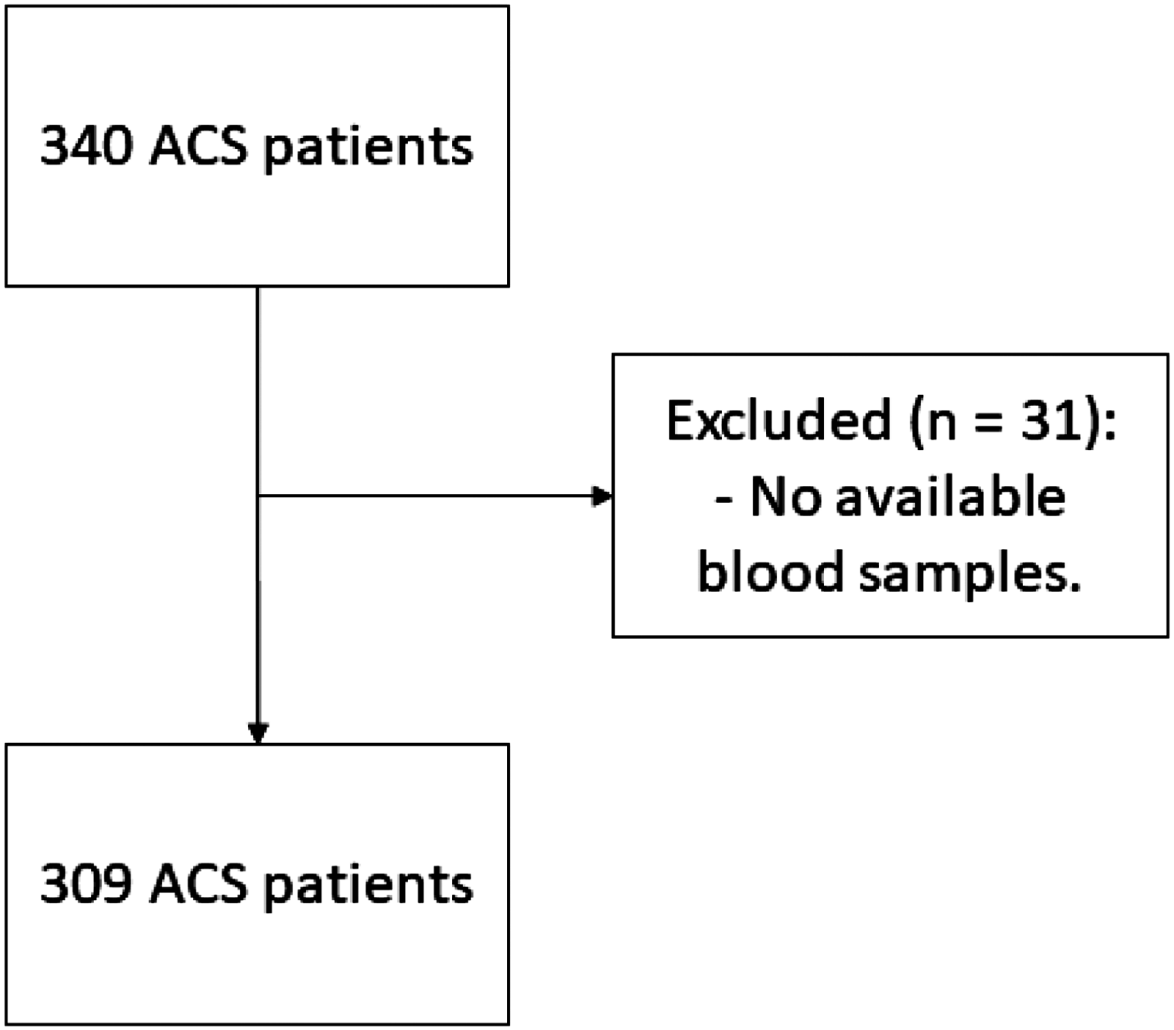
Flowchart of study participants. ACS, acute coronary syndrome.

This analysis used data from patients with ACS admitted to Joan XXIII University Hospital in Tarragona, Spain. The design and methods of this study have been described in detail elsewhere (12). Briefly, from January 2011 to May 2013, ACS patients who underwent coronary angiography and followed until 2022 were included in this study. A detailed description of the definition of ACS can be found here (12). Patients that suffered a MI other than type 1, and those with foreign residency were excluded from this study. From the initial sample of 340 patients recruited, we excluded 31 participants without blood samples (Figure 1).

### 2.2. Definitions of outcomes

Information on clinical outcomes was obtained by contacting patients every year, and by analysis of the hospital's patient-management information system [MI (ICD-10-CM I21) and hospitalization for heart failure (HF) (ICD-10-CM I50)]. MACE was defined as the composite of MI, hospitalization for HF, and all-cause mortality. All-cause mortality included cardiac, vascular (such as stroke, acute pulmonary edema, non-ischemic fatal arrythmia) and non-cardiovascular causes.

### 2.3. Quantification of TCA cycle metabolites

Plasma aliquots derived from blood samples (containing EDTA) collected from patients during coronary angiography have been stored at −80°C until analysis at the Biobank of the Pere Virgili Health Research Institute.

Citrate, aconitate, isocitrate, succinate, malate, fumarate, α-ketoglutarate and d/l-2-hydroxyglutarate were quantified by stable-isotope dilution method using liquid chromatography coupled to tandem mass spectrometry. For sample preparation, 100 μl plasma were mixed with 500 μl of methanol containing the mix of internal standards. Samples were vortexed and centrifuged for 10 min at 15,000 rpm and 4°C. Supernatants were evaporated in a SpeedVac concentrator at 45°C. Samples were reconstituted with 100 μl of 0.9% formic acid and separated in two glass vials for their analysis. In the case of fumarate, α-ketoglutarate and 2-hydroxyglutarate determination, samples were diluted with acetonitrile. To separate citrate, aconitate, isocitrate, succinate and malate, samples were chromatographed on an Atlantis Premier BEH C18 AX 1.7 µm, 2.1 mm × 100 mm (Waters, Milford, MA, USA) and in junction with a UHPLC 1290 Infinity II (Agilent Technologies, Santa Clara, CA, USA). The gradient consisted of 100% A for 1.4 min, to 40% A and 0% B at 1.5 min, to 17% A and 23% B at 3.51 min, to 0% A and 40% B at 4.00 min, kept 0% A and 40% B for.0.3 min, to 5% A and 95% B at 4.4 min and 5% A and 95% B for 0.1 min. Mobile phase A was composed of 0.9% formic acid in water, mobile phase B was 0.9% formic acid in acetonitrile and mobile phase C was 50 mm ammonium formate and 0.9% formic acid in water. Flow rate was kept constant at 0.35 ml/min, and the column manager was set at 30°C for the duration of the sequence. To separate fumarate, α-ketoglutarate and 2-hydroxyglutarate, samples were chromatographed on an ACQUITY UPLC BEH Amide, 1.7 µm, 2.1 mm × 150 mm (Waters, Milford, MA, USA) and in junction with a UHPLC 1290 Infinity II (Agilent Technologies, Santa Clara, CA, USA). The gradient consisted of 80% A for 2 min, to 50% A at 7 min, to 80% A at 7.1 min and 80% A for 2.9 min. Mobile phase A was composed of 10 mm ammonium acetate 0.05% ammonium hydroxide; mobile phase B was acetonitrile. Flow rate was kept constant at 0.5 ml/min, and the column manager was set at 40°C for the duration of the sequence. Agilent QqQ/MS 6490 Series with an electrospray ionization probe operating in negative ion mode was used for mass spectrometric analysis. The source conditions were set at 55 psi for the nebulizer gas, 120°C for the gas temperature, 20 L/min for the gas flow, 350°C for the sheath gas temperature, 11 L/min for the sheath gas flow, 1,100 V for the capillary voltage, and 500 V for the nozzle voltage. Detailed information of the quantitative determination for each compound and the mass to charge ratio and retention time are shown in Supplementary Table S1 (13). Assay quality assurance was monitored by routine analysis of pooled quality control plasma. The metabolite identification confidence level according to the published criteria (14) is Level 1: confirmed structure with MS, MS^2^, RT and reference standard.

### 2.4. Covariates

During hospital admission, demographics, clinical risk factors, previous medical conditions and medication use were registered. Participants were considered having type 2 diabetes (T2D), dyslipidemia, or hypertension if they had previously been diagnosed and/or used medications such as antidiabetic, cholesterol-lowering, or antihypertensive agents. Body mass index (BMI) was estimated as weight divided by height squared (kg/m^2^). The Chronic Kidney Disease Epidemiology Collaboration creatinine equation was used to calculate eGFR (15).

### 2.5. Statistical analyses

Baseline characteristics of study participants are described as means and standard deviations (SD) or medians and interquartile ranges for quantitative variables, and percentages for categorical variables. Importantly, our study had no missing data, and complete data were available for all participants included in the analysis. A natural logarithmic transformation was performed to approximate a normal distribution of metabolites' concentrations. In the following analyses, metabolites were analyzed as both continuous variables [1-standard deviation (SD) (1-SD) increment in their ln-transformed levels] and by using tertiles.

A crude and 2 multivariable Cox proportional hazards models were used for time-to-event analysis to determine hazard ratios (HRs) and 95% confidence intervals (CIs) for MACE and all-cause mortality. We checked the proportionality assumption of Cox regression by examining the Schoenfeld residuals and found that the proportional hazards assumption was not violated.

The first multivariable Cox regression model (multivariable model 1) was adjusted for age, sex, BMI (kg/m^2^), smoking (never, current, or former), hypertension (yes or no), dyslipidemia (yes or no), T2D (yes or no), unstable angina (yes or no), acute ST-segment elevation myocardial infarction (yes or no), non-ST-segment elevation acute myocardial infarction (yes or no), statin medication (yes or no), beta-blockers (yes or no), oral antidiabetic medication (yes or no), insulin medication (yes or no), diuretics (yes or no), and aspirin (yes or no). The second multivariable model (multivariable model 2) was additionally adjusted for eGFR to further assess the effect of renal function. We also examined associations of eGFR with MACE and all-cause mortality fitting the multivariable model 1 and further adjusting this model with each TCA cycle metabolite. To account for multiple testing, we adjusted *P* values of the crude and multivariable models with the use of the Benjamini-Hochberg false discovery rate procedure. Spearman's correlation analyses were performed to investigate whether there was a relationship between circulating levels of TCA cycle metabolites and eGFR. To test the robustness of our results related to model's overfitting, Weibull survival regression modelling was used. Cubic splines were used to fit the Cox regression models in order to examine non-parametric associations. Tests for non-linearity involved conducting a likelihood ratio test to compare the model with only the linear term to the model with both the linear and cubic spline terms. These analyses were performed using Stata 14.1 (Stata Corp.), at a two-tailed *α* of 0.05.

The mediating role of eGFR on the relationship between plasma metabolites and MACE or all-cause mortality was tested under a counterfactual framework (16). By performing this analysis using the R package “medflex” (17), the total effect of metabolites on MACE risk can be decomposed into a natural direct effect of metabolites on MACE or mortality risk and a natural indirect effect of metabolites accounted by the effect of the putative mediators, in our case the eGFR. The 95% confidence intervals were derived using bootstrapping with 1,000 replications. The mediation analysis was performed using Poisson based Generalized Linear Models by splitting the follow-up time every time an event was observed [survSplit() function in the survival package] in such a way every time interval contains only one event and thereafter we estimated the effects of the model parameters (18) as Cox regression models are currently not implemented in “medflex”. To approximate more the Cox results we added a time term to the Poisson model (19). We included all variables from the multivariable model 1 as covariates to adjust for the mediating effect of eGFR on the associations between TCA cycle metabolites and clinical outcomes. The mediated proportion was calculated as natural indirect log-incident rate ratios (IRR) divided by total effect log-IRR. This analysis was performed using R version 4.2.0 (R Foundation for Statistical Computing, Vienna, Austria).

## 3. Results

### 3.1. Baseline characteristics of patients

Patients' characteristics are summarized in Table 1. The majority of them were men (71.2%) and the mean age and eGFR of patients were 64.9 years, and 81.3 ml/min/1.73 m^2^, respectively. The majority of them had hypertension (67.6%) and dyslipidemia (60.8%). The prevalence of T2D was 37.2%, while current and former smokers were 30.4% and 33.9%, respectively. Of all patients, 62.0% were admitted with NSTEMI, 22.0% with STEMI and 15.9% with unstable angina. The median (interquartile range) of metabolites' concentrations can also be seen in Table 1.

**Table 1:** Baseline characteristics of the patients in the cohort.

Characteristics	Patients (*N* = 309)
Age (years)	64.9 ± 12.3
**Sex, %**	
Women	28.8
Men	71.2
Body mass index, kg/m^2^	28.1 ± 4.0
eGFR (ml/min/1.73 m^2^)	81.3 (62.1–96.7)
Type 2 diabetes, %	37.2
Hypertension, %	67.6
Dyslipidemia, %	60.8
**Smoking, %**	
Never	35.6
Former	33.9
Current	30.4
**Discharge diagnostic, %**	
Unstable angina	15.9
STEMI	22.0
NSTEMI	62.0
**Medications, %**	
Statins	50.5
Beta-blockers	30.4
Aspirin	40.5
Diuretics	25.2
Oral antidiabetic agents	22.7
Insulin medication	8.7
**Metabolites**	
Succinate, µmol/L	3.26 (2.48–3.99)
Fumarate, µmol/L	0.86 (0.71–1.05)
Malate, µmol/L	6.15 (4.99–7.84)
Citrate, µmol/L	86.26 (70.76–104.20)
Aconitate, µmol/L	2.02 (1.65–2.48)
Isocitrate, µmol/L	2.61 (1.87–3.63)
α-Ketoglutaric acid, µmol/L	9.25 (7.61–11.34)
d/l-2-Hydroxyglutarate, µmol/L	0.58 (0.45–0.81)

Continuous data are presented as mean ± standard deviation or median (interquartile range), and categorical variables are presented as %.

eGFR, estimated glomerular filtration rate; STEMI, ST elevation myocardial infarction; NSTEMI, non-ST elevation myocardial infarction; TMAO, trimethylamine N-oxide; TMA, trimethylamine.

### 3.2. Associations with the risk of MACE

During a mean follow-up time of 6.7 years (SD = 3.6), there were 131 incident MACE cases. In multivariable analyses, eGFR was associated with incident MACE (Supplementary Table S2). After further adjustment for TCA cycle metabolites, these associations were not attenuated. The associations between TCA cycle metabolites and MACE risk are displayed in Table 2. In the crude model, the estimated HR for incident MACE reached significance in the highest, compared with the lowest tertile of plasma concentrations of malate, aconitate, isocitrate and d/l-2-hydroxyglutarate, and these significant associations remained when these metabolites were treated as continuous (per 1 SD increment). However, in the multivariable model 1, only isocitrate remained significant (HR per 1 SD increment: 1.25; 95% CI: 1.03, 1.50; *P* = 0.020), while this association did not remain significant after accounting for multiple comparisons or Weibull survival analysis (Supplementary Table S3). Further adjustment for eGFR attenuated the associations between isocitrate and MACE. Spearman's correlation analyses revealed significant negative correlations between the majority of TCA cycle metabolites and eGFR levels (Supplementary Figure S1). No evidence for non-linearity in the association between isocitrate and MACE was found (*P*-value = 0.507) (Supplementary Figure S2).

**Table 2:** Associations of baseline individual metabolites concentrations with the risk of MACE^a^.

		Tertiles of plasma metabolite concentrations					
Metabolite	T1	T2	T3	*P* trend	HR per 1 SD increment	*P* value	FDR-adjusted *P* value
**Succinate**							
Concentrations (µmol/L)	<2.66	2.66–<3.69	≥3.69				
Cases	41	40	50				
Crude model	Ref.	0.87 (0.56–1.35)	1.22 (0.81–1.84)	0.338	1.10 (0.92–1.33)	0.292	0.333
MV1	Ref.	0.66 (0.41–1.08)	0.93 (0.59–1.46)	0.904	0.97 (0.80–1.19)	0.801	0.958
MV2	Ref.	0.70 (0.43–1.13)	0.88 (0.56–1.40)	0.688	0.95 (0.78–1.16)	0.605	0.806
**Fumarate**							
Concentrations(µmol/L)	<0.76	0.76–<0.97	≥0.97				
Cases	36	47	48				
Crude model	Ref.	1.36 (0.88–2.12)	1.48 (0.96–2.28)	0.090	1.23 (1.03–1.47)	**0** **.** **019**	**0** **.** **030**
MV1	Ref.	1.14 (0.69–1.88)	0.88 (0.54–1.44)	0.472	0.98 (0.81–1.20)	0.865	0.958
MV2	Ref.	1.12 (0.69–1.80)	0.69 (0.41–1.16)	0.100	0.88 (0.71–1.09)	0.247	0.806
**Malate**							
Concentrations (µmol/L)	<5.35	5.35–<7.27	≥7.27				
Cases	36	45	50				
Crude model	Ref.	1.39 (0.90–2.14)	1.78 (1.14–2.76)	**0** **.** **011**	1.33 (1.12–1.59)	**0** **.** **001**	**0** **.** **002**
MV1	Ref.	1.04 (0.62–1.74)	1.12 (0.67–1.88)	0.632	1.09 (0.89–1.33)	0.416	0.958
MV2	Ref.	0.95 (0.57–1.56)	0.86 (0.51–1.46)	0.562	0.98 (0.79–1.21)	0.833	0.833
**Citrate**							
Concentrations (µmol/L)	<75.34	75.34–<97.52	≥97.52				
Cases	44	40	47				
Crude model	Ref.	0.95 (0.63–1.50)	1.22 (0.81–1.84)	0.321	1.17 (0.98–1.41)	0.087	0.116
MV1	Ref.	0.75 (0.46–1.22)	0.68 (0.41–1.13)	0.152	0.95 (0.76–1.19)	0.672	0.958
MV2	Ref.	0.73 (0.45–1.17)	0.64 (0.39–1.07)	0.103	0.92 (0.73–1.15)	0.446	0.806
**Aconitate**							
Concentrations (µmol/L)	<1.79	1.79–<2.26	≥2.26				
Cases	32	41	58				
Crude model	Ref.	1.44 (0.90–2.29)	2.59 (1.66–4.03)	**<0** **.** **001**	1.44 (1.21–1.72)	**<0** **.** **001**	**0** **.** **002**
MV1	Ref.	1.14 (0.68–1.91)	1.64 (0.96–2.83)	0.060	1.20 (0.99–1.47)	0.065	0.260
MV2	Ref.	1.06 (0.64–1.78)	1.42 (0.81–2.48)	0.192	1.07 (0.85–1.34)	0.569	0.806
**Isocitrate**							
Concentrations (µmol/L)	<2.11	2.11–<3.19	≥3.19				
Cases	32	43	56				
Crude model	Ref.	1.61 (1.02–2.56)	2.52 (1.62–3.92)	**<0** **.** **001**	1.47 (0.23–1.75)	**<0** **.** **001**	**0** **.** **002**
MV1	Ref.	1.22 (0.71–2.08)	1.50 (0.88–2.56)	0.131	1.25 (1.03–1.50)	**0** **.** **020**	0.160
MV2	Ref.	1.17 (0.69–2.00)	1.28 (0.73–2.24)	0.411	1.11 (0.90–1.38)	0.334	0.806
**α–Ketoglutaric acid**							
Concentrations (µmol/L)	<8.06	8.06–<10.36	≥10.36				
Cases	41	46	44				
Crude model	Ref.	1.12 (0.73–1.71)	1.15 (0.74–1.78)	0.540	1.01 (0.85–1.19)	0.910	0.910
MV1	Ref.	1.18 (0.72–1.93)	1.17 (0.72–1.91)	0.552	0.99 (0.81–1.22)	0.958	0.958
MV2	Ref.	1.15 (0.71–1.85)	1.08 (0.66–1.78)	0.787	0.97 (0.79–1.19)	0.781	0.833
**d/l-2-Hydroxyglutarate**							
Concentrations (µmol/L)	<0.49	0.49–<0.72	≥0.72				
Cases	35	46	50				
Crude model	Ref.	1.53 (0.99–2.37)	1.68 (1.09–2.58)	**0** **.** **030**	1.26 (1.07–1.49)	**0** **.** **005**	**0** **.** **010**
MV1	Ref.	1.25 (0.74–2.12)	1.14 (0.69–1.86)	0.803	1.02 (0.85–1.23)	0.802	0.958
MV2	Ref.	1.13 (0.67–1.91)	0.88 (0.51–1.49)	0.422	0.89 (0.72–1.10)	0.288	0.806

FDR, false discovery rate; MV, multivariable; Ref, reference; MACE, major adverse cardiovascular events.

Bold text indicates statistically significant *P* values. FDR-controlled adjustments were conducted by applying the method of Benjamini and Hochberg.

^a^ Values are HR (95% CI). A natural logarithmic transformation was applied to the raw values of individual metabolites. Cox regression analysis was used. MV1 adjusted for age, sex, body mass index (kg/m^2^), smoking, hypertension, dyslipidemia, type 2 diabetes, unstable angina, acute ST-segment elevation myocardial infarction, non-ST-segment elevation acute myocardial infarction, statin medication, beta-blockers, oral antidiabetic medication, insulin medication, diuretics, aspirin. MV2 additionally adjusted for estimated glomerular filtration rate.

### 3.3. Associations with the risk of all-cause mortality

During a mean follow-up period of 7.8 years (SD = 3.1), 90 deaths were recorded, of which 35 were specifically attributed to cardiovascular causes. eGFR was significantly associated with all-cause mortality in the multivariable models (Supplementary Table S2). The associations between TCA cycle metabolites and all-cause mortality are shown in Table 3. In the multivariable model 1, the estimated HR for all-cause mortality reached significance only in the highest, compared with the lowest, tertile of plasma concentrations of d/l-2-hydroxyglutarate [2.20 (95% CI: 1.21, 3.98; *P* trend = 0.009). Significant positive associations with mortality were observed for aconitate, isocitrate and d/l-2-hydroxyglutarate when were modeled continuously (per 1 SD) (HR: 1.41; 95% CI: 1.07, 1.84; *P* = 0.014, HR: 1.58; 95% CI: 1.23, 2.02; *P* < 0.001 and HR: 1.38; 95% CI: 1.14, 1.68; *P* < 0.001, respectively). The associations between these metabolites and mortality persisted and remained significant after sensitivity analysis (Supplementary Table S4). These associations remained significant after accounting for multiple comparisons. However, these associations did not remain significant after inclusion of eGFR into the Cox regression model 1. Also, there was no evidence of non-linearity in these associations (*P*-value >0.05) (Supplementary Figure S3).

**Table 3:** Associations of baseline individual metabolites concentrations with the risk of all-cause mortality^a^.

		Tertiles of plasma metabolite concentrations					
Metabolite	T1	T2	T3	*P* trend	HR per 1 SD increment	*P* value	FDR-adjusted *P* value
**Succinate**							
Concentrations (µmol/L)	<2.66	2.66–<3.69	≥3.69				
Cases	28	26	36				
Crude model	Ref.	0.81 (0.47–1.39)	1.19 (0.73–1.94)	0.451	1.10 (0.88–1.38)	0.404	0.461
MV1	Ref.	0.61 (0.33–1.10)	0.85 (0.48–1.52)	0.688	0.96 (0.73–1.25)	0.738	0.738
MV2	Ref.	0.62 (0.36–1.09)	0.76 (0.43–1.35)	0.421	0.90 (0.70–1.16)	0.435	0.597
**Fumarate**							
Concentrations(µmol/L)	<0.76	0.76–<0.97	≥0.97				
Cases	24	32	34				
Crude model	Ref.	1.40 (0.82–2.38)	1.61 (0.96–2.72)	0.079	1.36 (1.10–1.67)	**0** **.** **004**	**0** **.** **006**
MV1	Ref.	0.84 (0.45–1.58)	0.68 (0.35–1.30)	0.230	0.95 (0.74–1.21)	0.674	0.738
MV2	Ref.	0.66 (0.37–1.19)	0.42 (0.21–0.83)	0.015	0.76 (0.56–1.03)	0.077	0.298
**Malate**							
Concentrations (µmol/L)	<5.35	5.35–<7.27	≥7.27				
Cases	21	32	37				
Crude model	Ref.	1.78 (1.03–3.08)	2.17 (1.26–3.74)	**0** **.** **007**	1.54 (1.26–1.88)	**<0** **.** **001**	**0** **.** **002**
MV1	Ref.	1.01 (0.52–1.96)	0.96 (0.51–1.82)	0.870	1.12 (0.87–1.45)	0.376	0.601
MV2	Ref.	0.92 (0.50–1.70)	0.63 (0.32–1.23)	0.118	0.95 (0.73–1.24)	0.714	0.714
**Citrate**							
Concentrations (µmol/L)	<75.34	75.34–<97.52	≥97.52				
Cases	25	26	39				
Crude model	Ref.	1.21 (0.69–2.14)	1.88 (1.14–3.10)	**0** **.** **010**	1.36 (1.09–1.69)	**0** **.** **006**	**0** **.** **008**
MV1	Ref.	0.62 (0.32–1.19)	0.53 (0.28–1.03)	0.078	0.84 (0.62–1.13)	0.244	0.488
MV2	Ref.	0.62 (0.32–1.21)	0.53 (0.27–1.03)	0.075	0.79 (0.59–1.08)	0.138	0.298
**Aconitate**							
Concentrations (µmol/L)	<1.79	1.79–<2.26	≥2.26				
Cases	16	27	47				
Crude model	Ref.	1.98 (1.05–3.75)	4.26 (2.40–7.56)	**<0** **.** **001**	1.85 (1.48–2.29)	**<0** **.** **001**	**0** **.** **002**
MV1	Ref.	0.88 (0.41–1.89)	1.34 (0.65–2.74)	0.269	1.41 (1.07–1.84)	**0** **.** **014**	**0** **.** **037**
MV2	Ref.	0.73 (0.35–1.52)	1.00 (0.49–2.05)	0.666	1.12 (0.84–1.48)	0.448	0.597
**Isocitrate**							
Concentrations (µmol/L)	<2.11	2.11–<3.19	≥3.19				
Cases	13	29	48				
Crude model	Ref.	2.92 (1.50–5.70)	6.18 (3.31–11.54)	**<0** **.** **001**	1.98 (1.56–2.50)	**<0** **.** **001**	**0** **.** **002**
MV1	Ref.	1.21 (0.51–2.85)	1.87 (0.85–4.12)	**0** **.** **046**	1.58 (1.23–2.02)	**<0** **.** **001**	**0** **.** **004**
MV2	Ref.	1.03 (0.44–2.41)	1.47 (0.66–3.26)	0.157	1.25 (0.95–1.06)	0.106	0.298
**α-Ketoglutaric acid**							
Concentrations (µmol/L)	<8.06	8.06–<10.36	≥10.36				
Cases	28	32	30				
Crude model	Ref.	1.16 (0.69–1.93)	1.14 (0.68–1.92)	0.632	0.98 (0.80–1.20)	0.846	0.846
MV1	Ref.	1.33 (0.72–2.44)	1.02 (0.59–1.75)	0.951	0.92 (0.73–1.17)	0.509	0.678
MV2	Ref.	1.34 (0.74–2.42)	1.01 (0.59–1.71)	0.892	0.94 (0.74–1.19)	0.614	0.701
**d/l-2-Hydroxyglutarate**							
Concentrations (µmol/L)	<0.49	0.49–<0.72	≥0.72				
Cases	17	29	44				
Crude model	Ref.	1.96 (1.10–3.49)	3.27 (1.89–5.67)	**<0** **.** **001**	1.62 (1.34–1.95)	**<0** **.** **001**	**0** **.** **002**
MV1	Ref.	1.44 (0.77–2.70)	2.20 (1.21–3.98)	**0** **.** **009**	1.38 (1.14–1.68)	**0** **.** **001**	**0** **.** **004**
MV2	Ref.	1.44 (0.78–2.66)	1.71 (0.94–3.11)	0.117	1.16 (0.95–1.42)	0.149	0.298

FDR, false discovery rate; MV, multivariable; Ref, reference.

Bold text indicates statistically significant *P* values. FDR-controlled adjustments were conducted by applying the method of Benjamini and Hochberg.

^a^ Values are HR (95% CI). A natural logarithmic transformation was applied to the raw values of individual metabolites. Cox regression analysis was used. MV1 adjusted for age, sex, body mass index (kg/m^2^), smoking, hypertension, dyslipidemia, type 2 diabetes, unstable angina, acute ST-segment elevation myocardial infarction, non-ST-segment elevation acute myocardial infarction, statin medication, beta-blockers, oral antidiabetic medication, insulin medication, diuretics, aspirin. MV2 additionally adjusted for estimated glomerular filtration rate.

### 3.4. Mediation of the association between TCA cycle metabolites, MACE and all-cause mortality

The mediation analyses were performed to assess the mediating role of eGFR in the relationships of TCA cycle metabolites with MACE and all-cause mortality. Results from these analyses are displayed in Table 4. A substantial proportion (75%) of the total effect of isocitrate (per 1-SD increase) on risk for MACE was mediated through eGFR, with natural direct effect of IRR: 1.08 (95% CI: 0.90–1.28) and natural indirect effect of IRR: 1.27 (95% CI: 1.14–1.46). Similarly, significant indirect effects on all-cause of mortality were found for aconitate, isocitrate and d/l-2-hydroxyglutarate (per 1-SD increase in their respective concentrations).

**Table 4:** Medflex counterfactual mediation analysis for MACE and all-cause mortality incident rate ratios (IRR) per 1 SD increment in TCA metabolites concentrations; proportion mediated by eGFR with bootstrap 95% confidence intervals (1,000 iterations).

Metabolite	Effect	IRR	95% Lower CI	95% Upper CI	Proportion mediated
**MACE**					
Isocitrate	Natural direct effect	1.08	0.90	1.28	75%
	Natural indirect effect	1.27	1.14	1.46	
	Total effect	1.38	1.19	1.58	
**All-cause mortality**					
Aconitate	Natural direct effect	1.18	0.92	1.44	67%
	Natural indirect effect	1.42	1.24	1.72	
	Total effect	1.68	1.41	1.91	
Isocitrate	Natural direct effect	1.22	0.97	1.50	63%
	Natural indirect effect	1.43	1.23	1.73	
	Total effect	1.74	1.50	2.01	
d/l-2-Hydroxyglutarate	Natural direct effect	1.20	0.96	1.47	58%
	Natural indirect effect	1.32	1.20	1.46	
	Total effect	1.58	1.32	1.88	

MACE, major adverse cardiovascular events; TCA, tricarboxylic acid cycle; eGFR, estimated glomerular filtration rate; CI, confidence intervals.

The magnitude of mediation was 67% for aconitate, 63% for isocitrate and 58% for d/l-2-hydroxyglutarate, with natural direct effects of IRR: 1.18 (95% CI: 0.92–1.44), IRR: 1.22 (95% CI: 0.97–1.50) and IRR: 1.20 (95% CI: 0.96–1.47), respectively, whereas the natural indirect effects of IRR were: 1.42 (95% CI: 1.24–1.72), 1.43 (95% CI: 1.23–1.73) and 1.32 (95% CI: 1.20–1.46), respectively.

## 4. Discussion

Of the 8 plasma TCA cycle metabolites measured at baseline, higher concentrations of isocitrate were consistently associated with MACE and all-cause mortality, while these associations became insignificant after adjustment for eGFR. Similar results were obtained for aconitate and d/l-2-hydroxyglutarate, that were found positively associated with all-cause mortality, while eGFR substantially attenuated these associations. Notably, eGFR fully mediated all above associations.

Recently, we analyzed trimethylamine-N-oxide (TMAO) and its precursors with risk of cardiovascular events in this population and demonstrated that both TMAO and dimethylglycine were positively associated with MACE (12). Furthermore, eGFR fully and partially mediated the associations of TMAO and dimethylglycine with MACE, respectively. Our results extend these findings which might be of interest in scaling back the clinical meaning of performing such expensive determinations in the attempt of finding robust predictors of adverse outcomes after ACS.

The mitochondrial TCA cycle function in heart and skeletal muscle is to oxidize the acetyl group of acetyl-CoA and to generate reducing equivalents (NADH**2**), which are used for the re-synthesis of ATP (20). Dysregulations of the TCA cycle have been associated to oxidative stress that could induce myocardial dysfunction and pro-inflammation (7, 21). Dysfunctional mitochondria can initiate the intrinsic mechanism of apoptosis of cardiomyocytes (22). In fact, this mechanism plays an important role on cardiac remodeling, when apoptotic and necrotic cardiomyocytes are replaced by fibroblasts in process of heart scarring induced worst prognosis (23). Furthermore, accumulation of TCA metabolites in different biological samples has been associated with hypertension (24), T2D (25), overweight and unfavourable lipidemic profile (26).

To our knowledge, our study is the first conducted in ACS patients from the Mediterranean region, with a long follow-up and demonstrated that patients with elevated plasma concentrations of isocitrate, had a higher risk of developing MACE after adjustment for several traditional cardiovascular risk factors and medications. Elevated plasma isocitrate levels may reflect mitochondrial nicotinamide adenine dinucleotide phosphate (NADP)+-isocitrate dehydrogenase inactivity. This enzyme catalyzes the oxidative decarboxylation of isocitrate producing a-ketoglutarate, regulates cardiomyocyte energy and redox status potentially contributing to cardiomyopathy (27). Shiomi et al. (28) conducted an experimental study in rabbits to find possible novel biomarkers that predict CVD and atherosclerosis. Coronary arteries from 363 rabbits were examined and classified concerning severity, using the ratio of the surface area of the atherotic lesions to the surface area of the entire lumen. The rabbits with severe atherosclerosis had significantly higher levels of isocitrate. Arterial stiffness, associated with atherosclerosis also, is strongly associated with an increase in the likelihood of a future new onset cardiovascular events, disease recurrence, or progression of CVD (29). Haam et al. (30) reported an association of elevated urine levels of isocitrate in 330 Korean patients with increased arterial stiffness. In a previous study conducted among 2,324 patients who underwent coronary angiography significant metabolic differences were found between patients with and without coronary artery disease (31). The TCA cycle was detected to be down-regulated and significantly associated with CVD. A recent case-control study conducted by Watany et al. (32) included 120 ACS patients and 120 healthy controls. They analyzed mitochondrial isocitrate dehydrogenase (IDH2) genetic isoforms and their variations were found to be an independent risk factor for acute myocardial infarction. This indicated a potential mechanism where higher levels of isocitrate due to this enzymatic dysfunction can induce an accumulation of intracellular free radicals mediating inflammation, lipid peroxidation and vascular dysfunction.

The two stereoisomers of 2-hydroxyglutarate are normally converted to 2-oxoglutarate in the mitochondrial matrix, to be metabolized by the TCA cycle. Findings from few observational studies suggest that aconitate, hydroxyglutarate (7) and 2-oxoglutarate (33) accumulation may indicate a disease state and suggest their potential clinical utility as biomarkers of metabolic stress or metabolic remodeling in disease. In addition, increased urine isocitrate and hydroxymethylglutarate concentrations were independently associated with brachial-ankle pulse wave velocity (34). Our study is the first to demonstrate that higher plasma concentrations of aconitate and d/l-2-hydroxyglutarate were associated with all-cause mortality. These metabolites were recently associated with higher risk of atrial fibrillation and HF in an elderly Mediterranean population (7).

Notably, eGFR substantially mediated these associations offering new insight into understanding the relationship between TCA metabolites, renal function, and adverse outcomes in ACS. Of note, we found negative correlations between isocitrate, aconitate and hydroxyglutarate with eGFR. A previous study also found negative correlations between plasma isocitrate and eGFR in CKD patients (35), and in another study a reduced urinary excretion of aconitate was observed in CKD (10). The involvement of TCA cycle metabolites in kidney diseases is well known (9). The renal function of our study population was mainly in a clinically normal range. CKD, also known as chronic kidney failure, is defined for patients younger than 40 years by eGFR below 75 ml/min/1.73 m^2^; between 40 and 65 years by 60 ml/min/1.73 m^2^; for older than 65 years without albuminuria or proteinuria by eGFR below 45 ml/min/1.73 m^2^ (36). Since the eGFR range in our sample was close to what is considered a normal renal function (age 64.9 ± 12.3, eGFR 62.1–96.7) it is reasonable to postulate that dysregulation of TCA cycle metabolism has preceded the development of kidney disease (9). Given that declined renal function is presented after ACS and is one of the main risk factors for cardiovascular outcomes and mortality (37), our results suggest that TCA cycle metabolites are associated with higher risk of clinical outcomes following ACS possibly due to renal decline. The causative mechanisms by which impaired kidney function contributes to cardiovascular diseases are not fully elucidated. CKD itself is considered a risk factor for ventricular and vascular remodeling and may serve to exacerbate the effects of other risk factors. Other possible mediators are: oxidative stress, inflammation, increased leptin, and/or activation of the renin-angiotensin system induced by renal impairment (38). eGFR emerges as practical and cost-effective marker that can be readily utilized for risk stratification in clinical practice. While adjusting for eGFR may reduce the direct prognostic significance of TCA metabolites, it is crucial to recognize the importance of profiling these metabolites for better understanding of underlying mechanisms with potential future implications in the management of ACS.

In addition to TCA cycle metabolites, other molecules such as soluble LOX-1, Cathepsin S, and LDL-electronegativity have also emerged as promising biomarkers for the prediction of adverse events in ACS (39–41), offering valuable insights into endothelial dysfunction, proteolytic activity, lipoprotein characteristics, and atherosclerosis-related processes. By integrating these biomarkers with the analysis of TCA cycle metabolites, a more comprehensive picture of the pathophysiology and risk assessment in ACS can be achieved.

### 4.1. Strengths and limitations

The main strengths of this study include its prospective design with a long-term follow-up, and without drop-outs and the analysis of several TCA cycle metabolites providing a broader understanding of the metabolic processes related to TCA and clinical outcomes. With regard to limitations, the findings of the present study cannot be generalized to younger adults and individuals with clinical features other than ACS. Furthermore, residual and unmeasured confounding may exist. Replication of the findings in an independent cohort study would strengthen the study conclusions.

## 5. Conclusions

In conclusion, we found that higher plasma TCA cycle metabolites including isocitrate, aconitate and d/l-2-hydroxyglutarate were associated with risk of MACE and all-cause mortality in ACS patients. Furthermore, mediation analyses demonstrated that eGFR is a strong mediator in these associations. While we acknowledge the potential confounding effect of eGFR, we undertook appropriate adjustments in our analyses, which enhanced the robustness of our findings. The identification of eGFR as mediator sheds light on the connection between TCA metabolites and adverse events in ACS. These findings underscore the significance of considering both TCA metabolites and renal function as conjunctive targets in the prevention of ACS complications.

## Data Availability

The raw data supporting the conclusions of this article will be made available by the authors, without undue reservation.
