# Supplementary material for: TCA cycle metabolites associated with adverse outcomes after acute coronary syndrome: mediating effect of renal function
Source: Front Cardiovasc Med. 2023 Jun 27;10:1157325. doi: 10.3389/fcvm.2023.1157325 (PMC10333508; doi:10.3389/fcvm.2023.1157325)

**Supplementary Table S1.** List of TCA metabolites identified in the LC-MS/MS method.

| Metabolite | HMDB_ID | Retention time (min) ^a and b^ | Precursor ion (m/z) | Product ion (m/z) |
| --- | --- | --- | --- | --- |
| Citric acid | HMDB00094 | 2.53^a^ | 191.0  191.0 | 110.9  87.0 |
| Isocitric acid | HMDB00193 | 1.46 ^a^ | 191.0  191.0 | 110.9  172.8 |
| Aconitic acid | HMDB00072 | 5.59 ^a^ | 173.0  173.0 | 85.0  110.0 |
| Malic acid | HMDB00156 | 1.35 ^a^ | 133.0  133.0 | 114.9  70.9 |
| Succinic acid | HMDB00254 | 2.36^a^ | 117.0  117.0 | 72.96  98.9 |
| Fumaric acid | HMDB00134 | 2.48^b^ | 115.0 | 70.8 |
| α-Ketoglutaric acid | HMDB00208 | 2.11^b^ | 145.0  145.0 | 101.0  56.9 |
| d/l-2-Hydroxyglutaric acid | HMDB00694 | 3.02^b^ | 147.0  147.0 | 56.8  128.9 |

^a^Separated on Atlantis Premier BEH C18 AX column and ^b^separated on ACQUITY UPLC BEH Amide column.

**Supplementary Table S2.** Associations of eGFR levels with the risk of MACE and all-cause mortality.

|  | **HR (95% CI)** | **P value** |
| --- | --- | --- |
| **MACE** |  |  |
| Model 1 | 0.98 (0.98-0.99) | 0.002 |
| Model 2 | 0.98 (0.98-0.99) | 0.002 |
| Model 3 | 0.98 (0.98-0.99) | 0.002 |
| Model 4 | 0.98 (0.98-0.99) | 0.005 |
| Model 5 | 0.98 (0.98-0.99) | 0.001 |
| Model 6 | 0.98 (0.98-0.99) | 0.017 |
| Model 7 | 0.98 (0.98-0.99) | 0.026 |
| Model 8 | 0.98 (0.98-0.99) | 0.002 |
| Model 9 | 0.98 (0.98-0.99) | 0.004 |
| **All-cause mortality** |  |  |
| Model 1 | 0.97 (0.96-0.98) | <0.001 |
| Model 2 | 0.97 (0.96-0.98) | <0.001 |
| Model 3 | 0.97 (0.95-0.98) | <0.001 |
| Model 4 | 0.97 (0.96-0.98) | <0.001 |
| Model 5 | 0.97 (0.96-0.98) | <0.001 |
| Model 6 | 0.97 (0.96-0.99) | <0.001 |
| Model 7 | 0.98 (0.96-0.99) | 0.001 |
| Model 8 | 0.97 (0.96-0.98) | <0.001 |
| Model 9 | 0.98 (0.96-0.99) | <0.001 |

Model 1 adjusted for age, sex, body mass index (kg/m^2^), smoking, hypertension, dyslipidemia, type 2 diabetes, unstable angina, acute ST-segment elevation myocardial infarction, non-ST-segment elevation acute myocardial infarction, statin medication, beta-blockers, oral antidiabetic medication, insulin medication, diuretics, aspirin; Model 2 additionally adjusted for succinate; Model 3 additionally adjusted for fumarate; Model 4 additionally adjusted for malate; Model 5 additionally adjusted for citrate; Model 6 additionally adjusted for aconitate; Model 7 additionally adjusted for isocitrate; Model 8 additionally adjusted for α-Ketoglutaric acid; Model 9 additionally adjusted for d/l-2-Hydroxyglutarate. Abbreviations: eGFR, estimated glomerular filtration rate.

**Supplementary Table S3.** Associations of baseline individual metabolites concentrations with the risk of MACE.

|  | **HR per 1 SD increment (95% CI)** | **P value** | **FDR-adjusted P value** |
| --- | --- | --- | --- |
| **MACE** |  |  |  |
| **Succinate** |  |  |  |
| MV1 | 0.98 (0.81-1.19) | 0.861 | 0.861 |
| MV2 | 0.96 (0.79-1.16) | 0.666 | 0.761 |
| **Fumarate** |  |  |  |
| MV1 | 0.96 (0.79-1.16) | 0.674 | 0.861 |
| MV2 | 0.85 (0.70-1.05) | 0.129 | 0.717 |
| **Malate** |  |  |  |
| MV1 | 1.06 (0.87-1.29) | 0.550 | 0.861 |
| MV2 | 0.95 (0.77-1.16) | 0.603 | 0.761 |
| **Citrate** |  |  |  |
| MV1 | 0.92 (0.74-1.13) | 0.415 | 0.861 |
| MV2 | 0.88 (0.71-1.08) | 0.227 | 0.717 |
| **Aconitate** |  |  |  |
| MV1 | 1.17 (0.95-1.43) | 0.135 | 0.540 |
| MV2 | 1.02 (0.82-1.28) | 0.819 | 0.819 |
| **Isocitrate** |  |  |  |
| MV1 | 1.21 (0.99-1.47) | 0.062 | 0.496 |
| MV2 | 1.07 (0.86-1.32) | 0.549 | 0.761 |
| **α-Ketoglutaric acid** |  |  |  |
| MV1 | 0.98 (0.80-1.20) | 0.839 | 0.861 |
| MV2 | 0.95 (0.78-1.16) | 0.628 | 0.761 |
| **d/l-2-Hydroxyglutarate** |  |  |  |
| MV1 | 1.03 (0.85-1.24) | 0.769 | 0.861 |
| MV2 | 0.89 (0.73-1.09) | 0.269 | 0.717 |

A natural logarithmic transformation was applied to the raw values of individual metabolites. Weibull regression analysis was used. MV1 adjusted for age, sex, body mass index (kg/m^2^), smoking, hypertension, dyslipidemia, type 2 diabetes, unstable angina, acute ST-segment elevation myocardial infarction, non-ST-segment elevation acute myocardial infarction, statin medication, beta-blockers, oral antidiabetic medication, insulin medication, diuretics, aspirin. MV2 additionally adjusted for estimated glomerular filtration rate.

Abbreviations: FDR, false discovery rate; MV, multivariable; MACE, major adverse cardiovascular events. FDR-controlled adjustments were conducted by applying the method of Benjamini and Hochberg.

**Supplementary Table S4.** Associations of baseline individual metabolites concentrations with the risk of all-cause mortality.

|  | **HR per 1 SD increment (95% CI)** | **P value** | **FDR-adjusted P value** |
| --- | --- | --- | --- |
| **MACE** |  |  |  |
| **Succinate** |  |  |  |
| MV1 | 0.99 (0.78-1.28) | 0.997 | 0.997 |
| MV2 | 0.97 (0.76-1.23) | 0.780 | 0.780 |
| **Fumarate** |  |  |  |
| MV1 | 0.90 (0.71-1.15) | 0.405 | 0.648 |
| MV2 | 0.75 (0.58-0.97) | **0.030** | 0.240 |
| **Malate** |  |  |  |
| MV1 | 1.08 (0.84-1.39) | 0.554 | 0.693 |
| MV2 | 0.94 (0.72-1.21) | 0.629 | 0.780 |
| **Citrate** |  |  |  |
| MV1 | 0.81 (0.61-1.07) | 0.135 | 0.270 |
| MV2 | 0.76 (0.58-1.01) | 0.061 | 0.244 |
| **Aconitate** |  |  |  |
| MV1 | 1.31 (1.00-1.72) | **0.046** | **0.122** |
| MV2 | 1.07 (0.81-1.42) | 0.645 | 0.780 |
| **Isocitrate** |  |  |  |
| MV1 | 1.44 (1.11-1.88) | **0.006** | **0.024** |
| MV2 | 1.17 (0.89-1.54) | 0.269 | 0.538 |
| **α-Ketoglutaric acid** |  |  |  |
| MV1 | 0.94 (0.73-1.20) | 0.607 | 0.693 |
| MV2 | 0.96 (0.75-1.22) | 0.746 | 0.780 |
| **d/l-2-Hydroxyglutarate** |  |  |  |
| MV1 | 1.39 (1.11-1.73) | **0.004** | **0.024** |
| MV2 | 0.18 (0.94-1.49) | 0.142 | 0.378 |

A natural logarithmic transformation was applied to the raw values of individual metabolites. Weibull regression analysis was used. MV1 adjusted for age, sex, body mass index (kg/m^2^), smoking, hypertension, dyslipidemia, type 2 diabetes, unstable angina, acute ST-segment elevation myocardial infarction, non-ST-segment elevation acute myocardial infarction, statin medication, beta-blockers, oral antidiabetic medication, insulin medication, diuretics, aspirin. MV2 additionally adjusted for estimated glomerular filtration rate.

Abbreviations: FDR, false discovery rate; MV, multivariable; MACE, major adverse cardiovascular events. Bold text indicates statistically significant P values. FDR-controlled adjustments were conducted by applying the method of Benjamini and Hochberg.

**Supplementary Figure S1.** Spearman’s correlation coefficients between plasma concentrations of TCA metabolites and estimated glomerular filtration rate (eGFR).

A


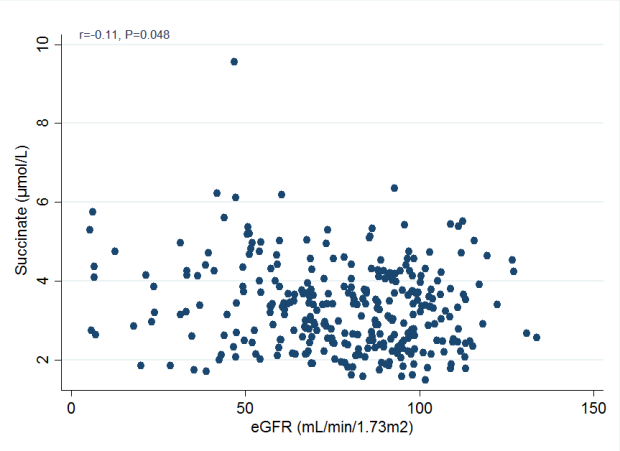


B


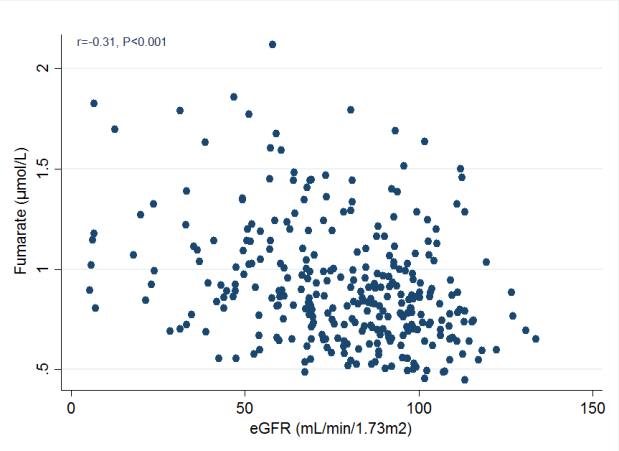


C


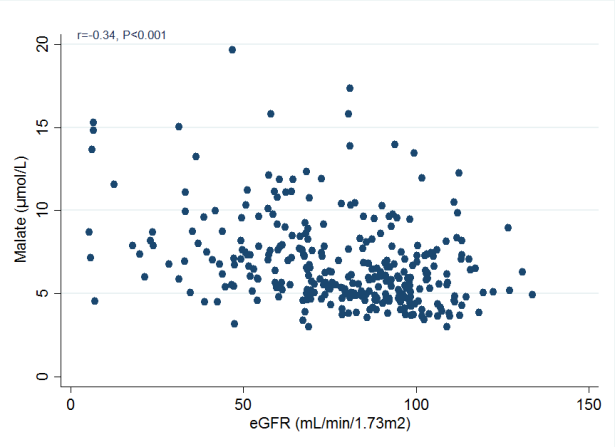


D


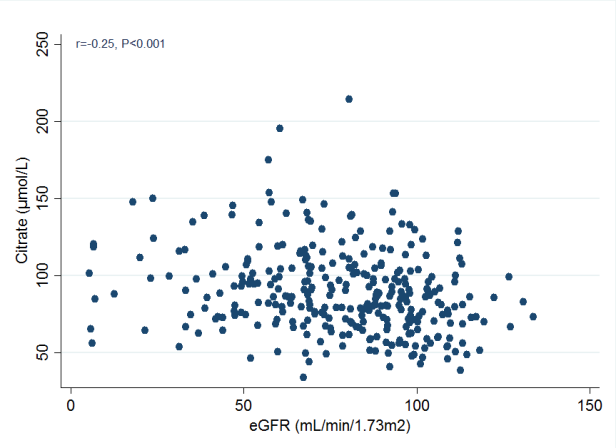


E


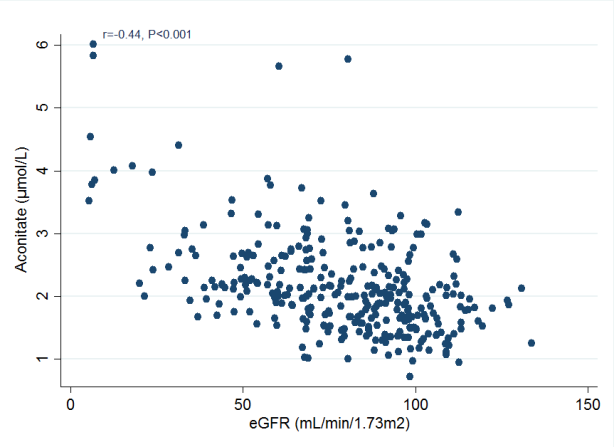


F


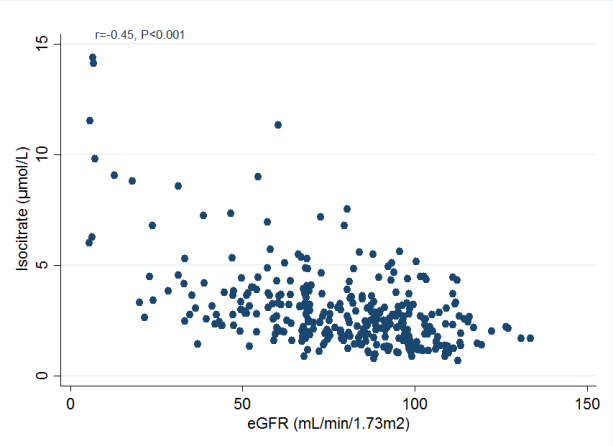


G


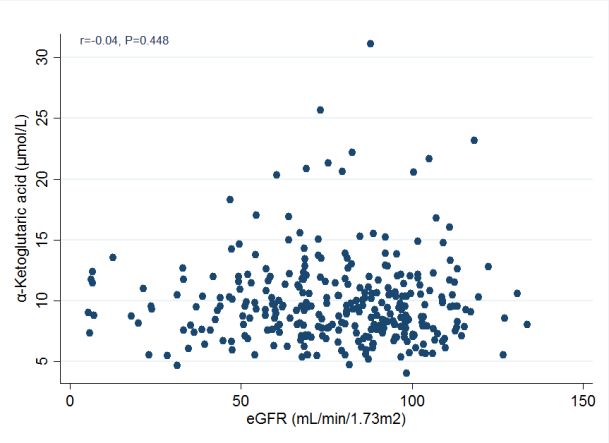


H


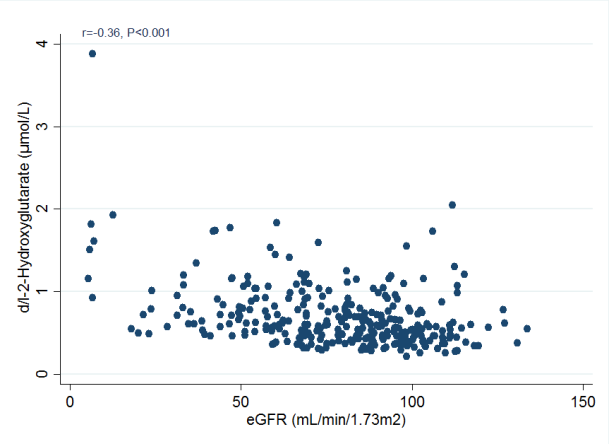


**Supplementary Figure S2.** Spline of concentrations of isocitrate and incident major adverse cardiovascular events. Dotted lines are 95% confidence intervals of the spline; horizon line is the reference.


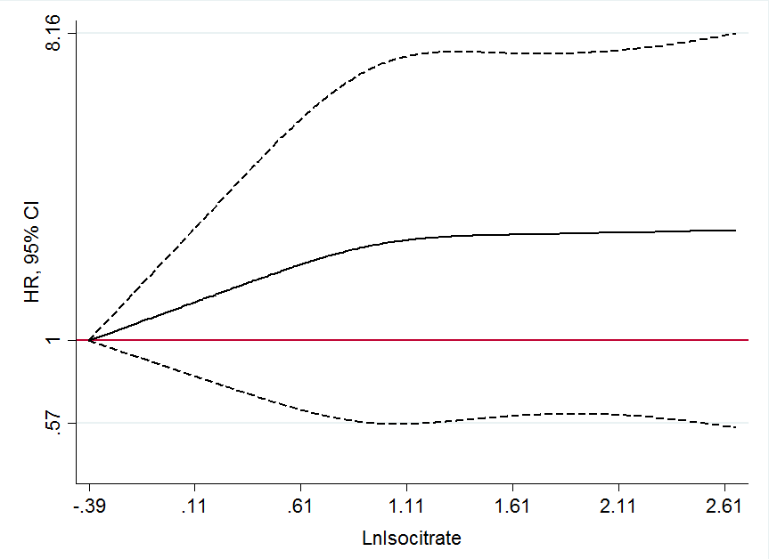


**Supplementary Figure S3.** Spline of concentrations of aconitate, isocitrate, d/l-2-hydroxyglutarate and all-cause mortality. Dotted lines are 95% confidence intervals of the spline; horizon line is the reference.


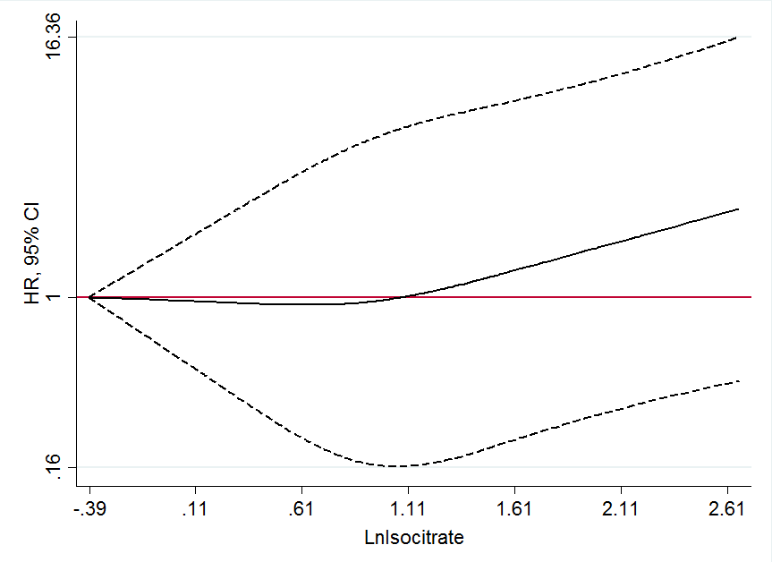


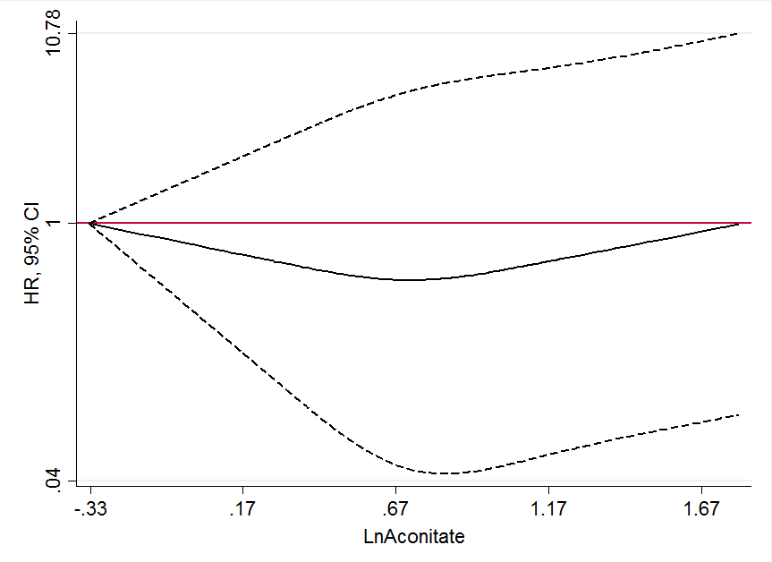


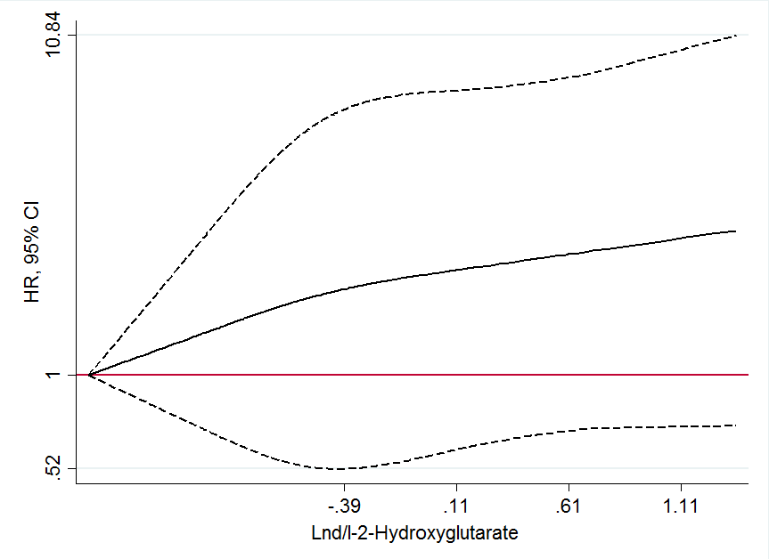

Supplement: Supplementary file 1 [file Table1.docx]
